# BMP3 inhibits TGFβ2-mediated myofibroblast differentiation during wound healing of the embryonic cornea

**DOI:** 10.1038/s41536-022-00232-9

**Published:** 2022-07-25

**Authors:** James W. Spurlin, Matthew R. Garis, Peter Y. Lwigale

**Affiliations:** Department of BioSciences, Rice University, Houston, TX USA

**Keywords:** Cell biology, Regeneration

## Abstract

Often acute damage to the cornea initiates drastic tissue remodeling, resulting in fibrotic scarring that disrupts light transmission and precedes vision impairment. Very little is known about the factors that can mitigate fibrosis and promote scar-free cornea wound healing. We previously described transient myofibroblast differentiation during non-fibrotic repair in an embryonic cornea injury model. Here, we sought to elucidate the mechanistic regulation of myofibroblast differentiation during embryonic cornea wound healing. We found that alpha-smooth muscle actin (αSMA)-positive myofibroblasts are superficial and their presence inversely correlates with wound closure. Expression of *TGFβ2* and nuclear localization of pSMAD2 were elevated during myofibroblast induction. *BMP3* and *BMP7* were localized in the corneal epithelium and corresponded with pSMAD1/5/8 activation and absence of myofibroblasts in the healing stroma. In vitro analyses with corneal fibroblasts revealed that BMP3 inhibits the persistence of TGFβ2-induced myofibroblasts by promoting disassembly of focal adhesions and αSMA fibers. This was confirmed by the expression of vinculin and pFAK. Together, these data highlight a mechanism to inhibit myofibroblast persistence during cornea wound repair.

## Introduction

Myofibroblasts are a specialized cell type that promote extracellular matrix (ECM) synthesis and remodeling^1^, regulate the tensile and contractile properties of healing tissue^2–5^, and facilitate rapid wound closure in tissue lesions^6^. Despite their significance during the initial stages of wound healing, persistence of myofibroblasts leads to fibrotic scarring in the affected tissues of many organs, including the heart^7,8^, lung^9–11^, skin^12–14^, kidney^15–17^, and eye^18–20^. In the cornea, persisting myofibroblasts have decreased levels of corneal crystallin proteins^21^ and synthesize high levels of disorganized ECM^22^, both of which obstruct light transmission required for visual acuity^20^. Therefore, spatiotemporal regulation of myofibroblasts in wounds is critical to promote non-fibrotic healing and tissue remodeling^2,23,24^.

Transforming growth factor β (TGFβ) signaling plays an important role in fibrosis by promoting the pro-fibrotic response and deposition of ECM^25–27^. The TGFβ superfamily consists of three isoforms of TGFβ (TGFβ1, 2, and 3), activins, growth differentiation factors (GDFs), and at least 20 isoforms of bone morphogenic protein (BMP). Signaling by the TGFβ superfamily is involved in multiple cellular processes during development, homeostasis, injury, and disease^28,29^. The three TGFβ isoforms signal by binding to serine/threonine kinase receptors including type I (ALK4, ALK5, ALK7) and type II (TGFβRII). This leads to phosphorylation of SMAD2/3 (pSMAD2/3), which forms a complex with SMAD4 and is translocated into the nucleus where it regulates TGFβ downstream targets, including profibrotic genes^30,31^. In contrast, BMP7 signaling via pSMAD1/5/8 prevents TGFβ1-induced fibrosis^32,33^. BMPs bind to type I (ALK1, ALK2, ALK3, ALK6) and type II (BMPRII, ActRII, ActRIIB) receptors to phosphorylate SMAD1/5/8. The pSMAD1/5/8 forms a complex with SMAD4, and it is translocated to the nucleus where it regulates BMP targets such as the inhibitors of differentiation (Id) genes, which have been shown to suppress TGFβ1-mediated fibrosis^34–36^. ActRII and ActRIIB also function as receptors for activins and myostatin to activate pSMAD2/3^31,37^.

Transduction of canonical TGFβ1 and TGFβ2 signaling mediated by pSMAD2 is a potent inducer of myofibroblast differentiation in tissues^38,39^. Concurrent activation of integrins promotes phosphorylation of focal adhesion kinase (FAK) in cells stimulated with TGFβ1 and cultured on a dense or stiffened ECM^40–44^. TGFβ1 drives neo-expression of αSMA via canonical SMAD and non-canonical integrin-FAK-ROCK signaling pathways^45,46^. αSMA is later incorporated into stress fibers that are anchored to the focal adhesion complexes on the cytoplasmic domains of the integrin receptors^33,34^. In addition, myofibroblasts secrete and interact with a wound-specific fibronectin splice variant, EDA-FN, which further activates TGFβ1^47–49^ and integrin signaling^48^. These ECM-cell interactions establish a positive feedback loop that enables myofibroblast persistence in tissues^38^.

Targeting canonical TGFβ signal transduction in tissues is one of the most prevalent approaches for inhibiting myofibroblasts and mitigating fibrotic progression in many tissues. These include sequestering TGFβ1 or TGFβ2 ligands^50–53^, inhibition of pSMAD2 signal transduction^54,55^, and addition of antifibrotic recombinant proteins such as BMP7 that stimulate pSMAD1/5/8 signal transduction^33,56,57^. Indeed, exogenous expression of BMP7 in cornea wounds suppresses TGFβ signaling and myofibroblast differentiation^18,58–60^.

Fetal tissues, including the skin, bone, heart, central nervous system, and cartilage, have a remarkable ability to heal without scaring^61–67^. Moreover, fetal wound healing experiments reveal that embryonic tissues have intrinsic properties that promote non-fibrotic repair^68,69^, and their regenerative capacity is not just attributed to the fetal environment. The most conserved observations in various fetal wound healing models include low levels of inflammation, rapid deposition of highly organized ECM, and transient populations of repair myofibroblasts^70–73^. However, such pro-regeneration healing mechanisms are restricted by gestational age^65^, and drastically wane after birth. Therefore, embryonic injury models can provide insight into how cells endogenously regulate myofibroblast differentiation to modulate fibrosis.

Our previous studies using the embryonic cornea wound healing model revealed that transient expression αSMA is coupled with non-fibrotic healing^74,75^. Like other tissues capable of fetal regeneration, non-fibrotic healing in the cornea is restricted to embryonic stages. Stromal injury in post-natal chick corneas leads to upregulation of TGFβ1, TGFβ2, and pSMAD2 concomitant with production of ECM proteins and myofibroblast differentiation^76,77^. Here, we utilize the embryonic cornea wound healing model to identify the mechanistic regulation of myofibroblast induction and rapid suppression during non-fibrotic repair in the cornea. We test the hypothesis that in the embryonic cornea, myofibroblasts are induced by TGFβ/pSMAD2 signaling and that their transient phenotype is due to suppression by BMP/pSMAD1/5/8.

## Results

### Myofibroblast differentiation during embryonic cornea wound healing correlates with TGFβ signaling

**Fig. 1:**
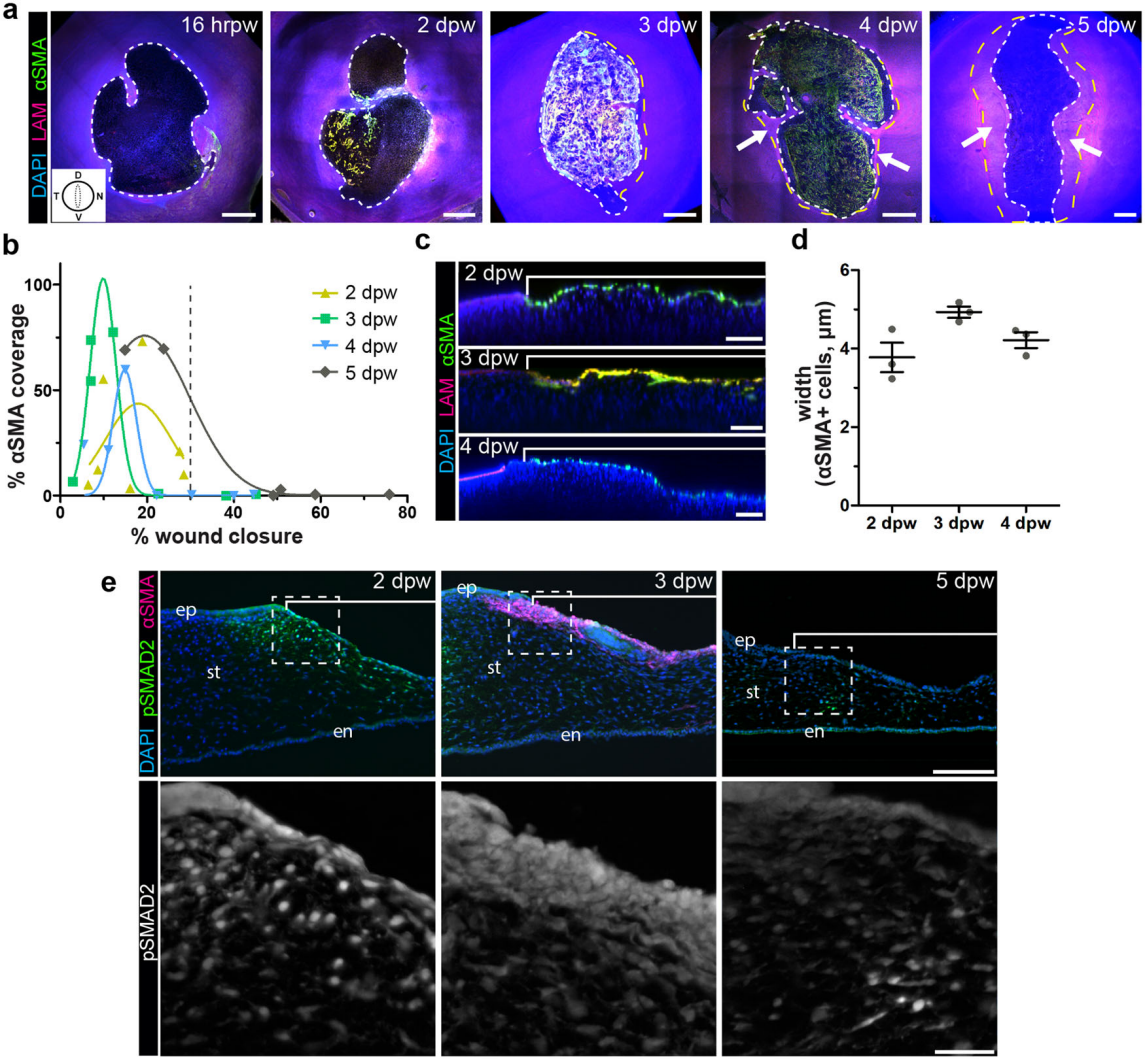
Induction of myofibroblast during wound healing of the embryonic cornea correlates with pSMAD2 activation. **a** Embryonic corneas wounded at E7 were collected between 16 hrpw and 5 dpw and analyzed via scanning confocal microscopy. All corneas were oriented as indicated in the inset with the dotted line representing the region of the wound (D, dorsal; V, ventral; T, temporal; N, nasal). Samples were immunostained for epithelial basement membrane protein laminin (magenta, LAM) to identify subepithelial wound penetration, α-smooth muscle actin (green, αSMA) to label myofibroblasts, and counterstained with DAPI (blue) to identify all nuclei. Front of healing epithelium (white dotted line) and previously regenerated epithelium (yellow dotted line) were identified based on laminin staining. Scale bar: 400 µm. **b** Quantification of the magnitude of myofibroblast differentiation in cornea wounds. The total area of denuded stroma was divided by cumulative αSMA-positive area to calculate percentage of wound covered by myofibroblasts. Myofibroblasts were not detected in cornea wounds that were >30% re-epithelized (*N* = 3–7 independent samples were analyzed at each time point). **c** Optical reconstructions of confocal scans showing the depth of αSMA-positive cells in the stromal wounds. **d** Quantification of depths at various time points. Analysis was restricted to corneas containing myofibroblasts in the denuded stroma (*N* = 3, 2–4 dpw; *N* = 2, 5 dpw). Data are shown as mean ± SEM. Scale bar: 100 µm. **e** Histological sections from 2, 3, and 5 dpw corneas were immunostained for pSMAD2 (green and gray scale) to highlight regions with TGFβ signal transduction, αSMA (magenta) for myofibroblast differentiation, and counterstained with DAPI (blue). Brackets indicate the region of the wound. Scale bars: 100 µm, insets: 25 µm. ep epithelium, st stroma, en endothelium.

We previously reported a transient population of myofibroblasts during wound healing of the embryonic cornea^74^. However, their spatiotemporal occupancy of the cornea wound was not investigated. We therefore first analyzed the distribution of αSMA-positive myofibroblasts in whole-mount corneas at various time points during wound healing. We confirmed the presence of stromal wounds by the absence of laminin staining, which indicated disruption of the corneal epithelial basement membrane (Fig. 1a). αSMA-positive cells were evident in the wound by 2 days post wound (dpw) and reached maximum coverage at 3–4 dpw. We also observed that downregulation of αSMA corresponded with corneal re-epithelialization at 4 and 5 dpw (Fig. 1a, arrows). Intriguingly, no myofibroblasts were detected in cornea wounds with greater than 30% epithelial closure, regardless of the time post-injury (Fig. 1b). In rare cases, some corneas at 5 dpw exhibited delayed epithelial closure (>30% healed) and still maintained αSMA positive cells in the stroma. Furthermore, cross-sections of cornea wounds revealed that the αSMA-positive cells occupied only the anterior region of the stroma, within the depth of 4–5 µm (Fig. 1c, d), which is in contrast to post-natal chick cornea wounds that undergo extensive fibrosis in the stroma^76,77^. The αSMA-positive cells also stained positive for vimentin (Supplementary Fig. 1), indicating their origin from the local mesenchyme of the cornea stroma and that they probably belong to the V-type myofibroblasts, which are present during early wound healing^78,79^. Together, these results indicate that transient differentiation of myofibroblasts in the embryonic cornea wound is superficial and inversely correlates with the re-epithelization.

To determine whether TGFβ signaling plays a role during the induction of αSMA-positive myofibroblasts, we stained histological sections from 2–5 dpw for pSMAD2. We observed robust nuclear localization of pSMAD2 in the stromal wound at 2 dpw, prior to the onset of αSMA expression (Fig. 1e). At 3 dpw, pSMAD2 staining was primarily localized in the anterior region of the stroma wound where the majority of cells expressed αSMA. By 5 dpw only a few stromal cells were positive for pSMAD2, which corresponded with the absence of αSMA in the corneal wound (Fig. 1e). These results indicate that induction of myofibroblast differentiation and their transient appearance respectively correspond with the activation and downregulation of TGFβ.

### TGFβ2 drives myofibroblast differentiation in corneal fibroblasts

**Fig. 2:**
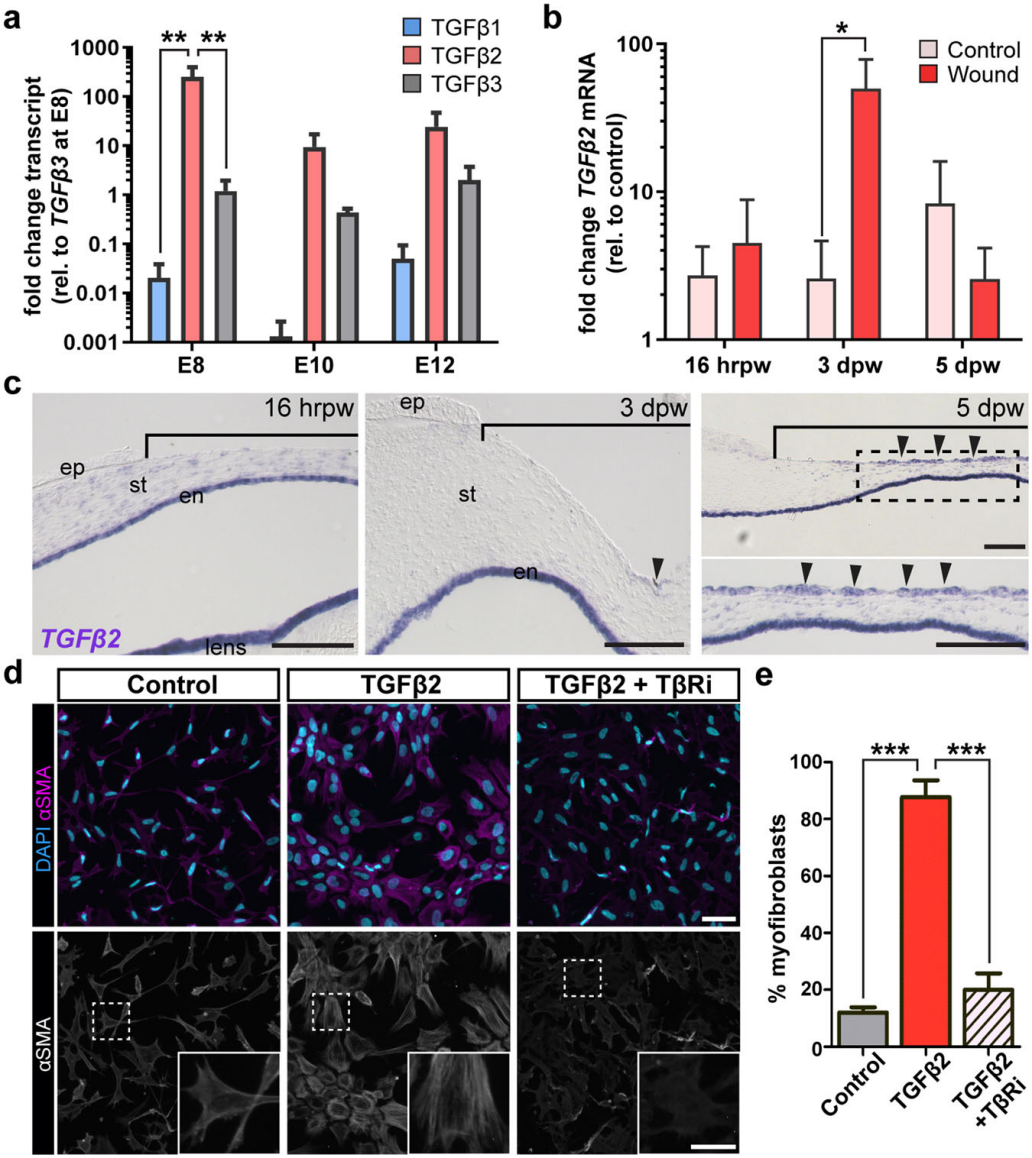
Expression of TGFβ2 transcripts and its role in myofibroblast differentiation. **a** qPCR analysis of *TGFβ1*, *TGFβ2* and *TGFβ3* transcript levels in E8, E10 and E12 control corneas (*N* = 3–4 independent samples). **b** qPCR analysis of *TGFβ2* transcript levels during wound healing in embryonic corneas. Levels were compared to each respective stage matched control at 16 hrpw, 3 dpw, and 5 dpw (*N* = 4 independent samples). **c** In situ hybridization for *TGFβ2* was performed on histological sections of 16 hrpw, 3 dpw and 5 dpw wounded corneas. Brackets indicate region of the wound. **d** Primary keratocytes isolated from E10 corneas were cultured in the absence (control) or presence of TGFβ2, and in the presence of TGFβ2 and SB431542 inhibitor (TβRi). Cells were immunostained for αSMA (magenta) and counterstained with DAPI. Myofibroblast phenotype was identified by αSMA-positive fibers (insets). **e** Quantification of myofibroblast differentiation. (*N* = 3 independent experiments; images were taken from 5 fields of each sample and the number of cells averaged; *N* = 535 cells control, *N* = 705 cells TGFβ2, and *N* = 718 cells TGFβ2 + TβRi). Data were assumed to be normally distributed and are shown as mean ± SEM. Two-way ANOVA with Bonferroni’s post-test (**a**), Student’s two-tailed, unpaired *t*-test (**b**) or One-way ANOVA with Tukey’s post-test (**e**) were performed. *p < 0.05, **p < 0.01, ***p < 0.001. Scale bars: 100 µm. Scale bars: 50 µm, inset: 20 µm. ep epithelium, st stroma, en endothelium.

Next, we investigated which isoforms of TGFβ are present in the cornea during wound healing. First, we examined the endogenous levels of expression in control corneas that correspond to 16 h post wound (hrpw, embryonic day [E]8), 3 dpw (E10), and 5 dpw (E12). qPCR analysis showed that *TGFβ2* transcripts were significantly elevated compared to *TGFβ1* and *TGFβ3* at E8 but decreased at E10 and E12 (Fig. 2a). Overall, *TGFβ1* was the least detected isoform (Fig. 2a). We confirmed by in situ hybridization that *TGFβ1* and *TGFβ3* were localized to the corneal epithelium, whereas *TGFβ2* was transiently expressed in the stroma, although it remained consistent in the corneal endothelium at these developmental stages (Supplementary Fig. 2, arrowheads). These results indicate that *TGFβ2* is the most abundant isoform, and its localization in the stroma, makes it a potential candidate for pSMAD2 induction and subsequent myofibroblast differentiation.

To determine the role *TGFβ2* during embryonic cornea wound healing, we assessed its transcript levels in wounded and stage matched controls. qPCR analysis revealed that *TGFβ2* was significantly upregulated at 3 dpw compared to controls (Fig. 2b). Next, we examined the localization of *TGFβ2* in the wounded corneas by section in situ hybridization. At 16 hrpw, *TGFβ2* was expressed in the stroma and corneal endothelium (Fig. 2c), consistent with E8 control corneas (Supplementary Fig. 2b). However, unlike controls where expression is subsequently downregulated in the stroma by E10 (Supplementary Fig. 2b), *TGFβ2* was maintained in the stroma at 3 and 5 dpw (Fig. 2c). qPCR analysis of *TGFβ1* and *TGFβ3* showed no significant enrichment during wound healing compared to stage-matched controls (Supplementary Fig. 3a, b). Interestingly, their levels of expression were elevated in the regenerating corneal epithelium at 3 and 5 dpw (Supplementary Fig. 3c, arrowheads). These results further suggest that TGFβ2 plays a role in myofibroblast induction during the healing of incisional corneal wounds in embryonic chicken corneas.

Next, we tested whether TGFβ2 promotes myofibroblast induction in vitro. Primary keratocytes were isolated from E10 corneas and cultured for 72hrs with or without recombinant TGFβ2, and in the presence of TGFβ2 and SB431542 inhibitor (TβRi). Since primary keratocytes cultured in the presence of fetal bovine serum (FBS) transform into fibroblasts^80,81^, we will refer to the cells cultured in 0.5% FBS as corneal fibroblasts. Cells were immunostained for αSMA to quantify myofibroblast differentiation. In the presence of TGFβ2, cells expressed high levels of αSMA, which appeared as stress fibers, thus indicating myofibroblast differentiation (Fig. 2d, e). In contrast, low levels of αSMA staining and no stress fibers were observed both in the absence of TGFβ2, and when cells were cultured in TGFβ2 and TβRi (Fig. 2d, e). Combined, these results indicate that TGFβ2 induces myofibroblast differentiation in corneal fibroblasts, suggesting it is a driver of this process during wound healing in the embryonic cornea.

### BMP signaling is upregulated during wound healing in embryonic corneas

**Fig. 3:**
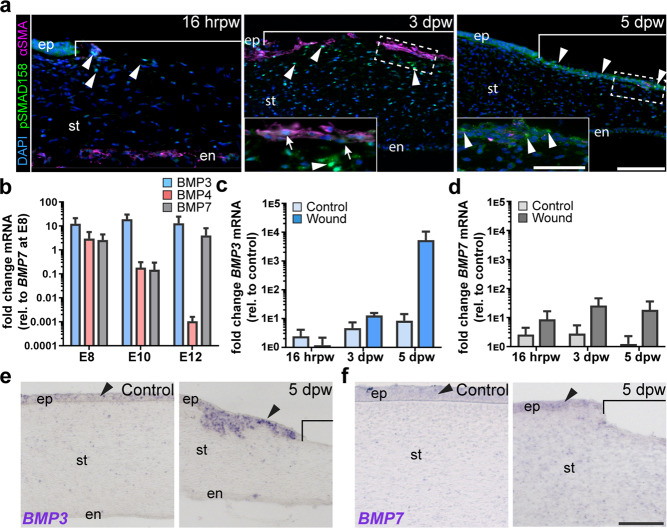
BMP3 and BMP7 are upregulated in healing embryonic cornea wounds. **a** Histological sections from 16 hrpw, 3 dpw and 5 dpw wounded embryonic corneas were immunostained with pSMAD1/5/8 (green) to highlight BMP signal transduction, αSMA (magenta) to label myofibroblasts, and counterstained with DAPI (blue) to identify all nuclei. pSMAD1/5/8 staining was localized to the nuclei of αSMA-negative cells (arrowheads), and a few αSMA-positive cells (arrows). Scale bars: 100 µm, inset: 50 µm. **b** qPCR analysis of transcript levels for *BMP3*, *BMP4* and *BMP7* (*N* = 3 or 4 independent samples). **c**, **d** *BMP3* and *BMP7* transcript levels were measured in wounded corneas at 16 hrpw, 3 dpw (*N* = 3 or 4 independent samples), and 5 dpw (*N* = 5 or 6 independent samples), comparing each time point to its respective stage-matched control. **e**, **f** In situ hybridization was performed on histological sections at 5 dpw and stage matched controls to reveal localization of for *BMP3* and *BMP7* transcripts. Brackets in **a**, **e** indicate region of the wound. Data were assumed to be normally distributed and are shown as mean ± SEM. Two-way ANOVA with Bonferroni’s post-test (**b**), Student’s two-tailed, unpaired *t*-test (**c**, **d**) were performed. *p < 0.05, **p < 0.01, ***p < 0.001. Scale bars: 100 µm. ep epithelium, st stroma, en endothelium.

BMP7 prevents TGFβ1-mediated keratocyte differentiation into myofibroblasts^82–85^. We hypothesized that BMP signaling may contribute to the transient myofibroblast phenotype in the healing embryonic cornea. First, we determined whether BMP signaling occurs during wound healing by immunostaining for pSMAD1/5/8 between 16 hrpw and 5 dpw. At 16 hrpw, only a few nuclei in the stroma were positive for nuclear pSMAD1/5/8 (Fig. 3a, arrowheads), and no αSMA-positive cells were detected in the anterior stroma. However, by 3 dpw, several pSMAD1/5/8-positive nuclei were distributed throughout the stroma and anterior surface of the wound (Fig. 3a and inset, arrowheads). At this time, only a few αSMA-positive cells stained positive for pSMAD1/5/8 (Fig. 3a, 3 dpw inset arrows). At 5 dpw, pSMAD1/5/8 was most prominent in the anterior stroma of the wound when αSMA staining was negligible (Fig. 3a, arrowheads). These results show that the spatiotemporal distribution of pSMAD1/5/8 corresponds with the absence of αSMA in the cornea wound, suggesting a potential role in its downregulation and transient incidence of myofibroblasts.

To determine which BMP isoforms are responsible for the activation of pSMAD1/5/8 during wound healing, we performed qPCR analysis on control corneas for *BMP3*, *BMP4*, and *BMP7*, which were previously observed in adult human corneas^86^. Our results revealed that transcripts for *BMP3* and, to a lesser extent *BMP4* and *BMP7*, were amplified endogenously between E8-E12 (Fig. 3b). Here, we focused on *BMP3* and *BMP7*, which were localized to the corneal epithelium during normal development (Supplementary Fig. 4). Compared to stage-matched controls, the transcript levels of both *BMP3* and *BMP7* were not significantly upregulated in wounded corneas when compared to stage matched controls at all the time points (Fig. 3c, d). Nonetheless, there was an increasing trend of *BMP3* and *BMP7* expression between 16 hrpw and 5 dpw. Therefore we examined their expression patterns by in situ hybridization at 5 dpw. *BMP3* expression was localized to the corneal epithelium and vividly expressed in the regenerating epithelium of the wound (Fig. 3e, arrowheads). *BMP7* was also expressed in the corneal epithelium, but it was modestly elevated during re-epithelialization (Fig. 3f, arrowheads). Combined, these findings indicate that *BMP3* and *BMP7* are upregulated during re-epithelization of the embryonic cornea wound and spatiotemporally correlate with the activation of pSMAD1/5/8. Suggesting that BMP3 and BMP7 secreted from the healing cornea epithelium may play a role in suppressing the αSMA-positive myofibroblast phenotype in the anterior stroma of the wound.

### BMP3 antagonizes TGFβ2-mediated myofibroblast phenotype in corneal fibroblasts

**Fig. 4:**
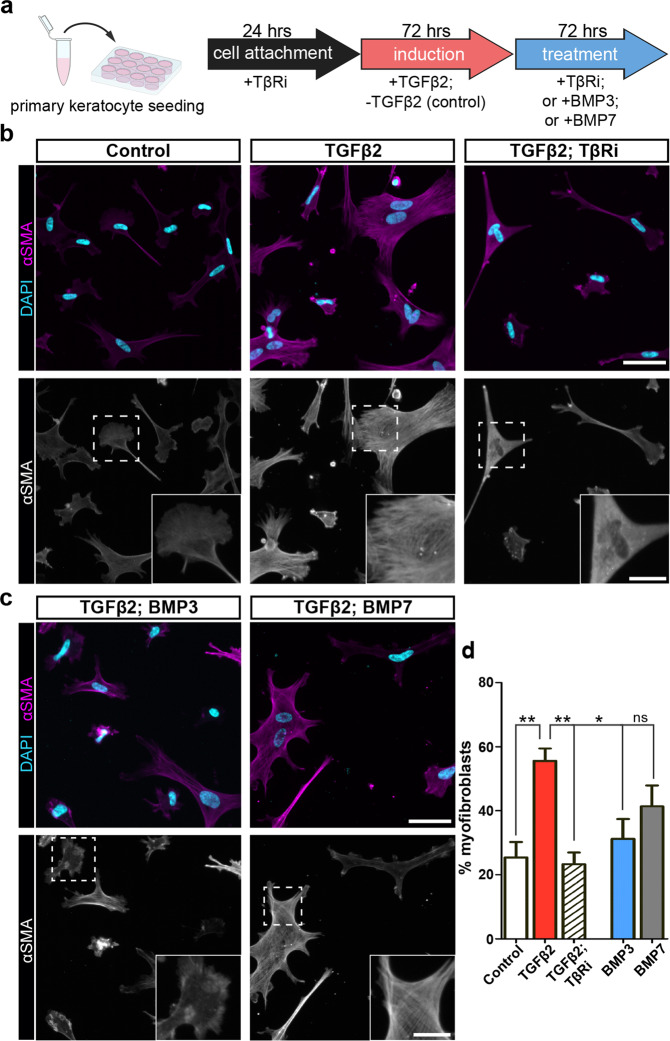
BMP3 antagonizes induced myofibroblast phenotype in isolated embryonic keratocytes. **a** Schematic showing how primary keratocytes were cultured and treated in two phases of the experiment. Cells were seeded in the presence of SB431542 inhibitor (TβRi) for 24 h. In phase 1, cells were either cultured uninduced (control) or induced with TGFβ2 for 72 h. During phase 2, induced cells were either untreated or treated with TβRi, BMP3, or BMP7. **b** At the end of the experiment, uninduced (control) cells showed low levels of αSMA staining compared to TGFβ2-induced but untreated cells, which stained vividly for αSMA stress fibers (insets). **c** Few TGFβ2-induced cells treated with TβRi showed high levels of αSMA, but they did not have the stress fibers (insets). Similar reduction in myofibroblast phenotype was observed in TGFβ2-induced cells treated with BMP3, and to a lesser extent with BMP7. **d** Quantification of the percentage of myofibroblasts present in culture observed in **b**, **c** (*N* = 4 independent experiments; images were taken from 5 fields of each sample and the number of cells averaged; *N* = 622 cells control, *N* = 226 cells TGFβ2, *N* = 363 cells TGFβ2 + TβRi, *N* = 282 cells TGFβ2 + BMP3, and *N* = 244 cells TGFβ2 + BMP7). Data were assumed to be normally distributed and are shown as mean ± SEM. One-way ANOVA with Tukey’s post-test (**d**) was performed. *p < 0.05, **p < 0.01, ns not significant. Scale bars: 50 µm, inset: 20 µm.

While BMP7 prevents myofibroblast differentiation in the adult cornea^84,85^, it is unclear if this activity is conserved in embryonic keratocytes. In addition, the role of BMP3 in this process is unknown. To test if BMP3 and BMP7 attenuate embryonic myofibroblast differentiation in vitro, primary keratocytes were induced with TGFβ2, then cultured in the presence of either TβRi, BMP3, or BMP7 (Fig. 4a).

Following induction, cells maintain αSMA-positive stress fibers even after removal of TGFβ2, thus the myofibroblast phenotype is stable on the time scale of this experiment (Fig. 4b, d). When induced myofibroblasts were treated with TβRi, they appeared fibroblastic, but lost the αSMA stress fibers (Fig. 4b, d). These results indicate that embryonic myofibroblasts are capable of disassembling αSMA stress fibers and losing the phenotype in vitro.

Next, we examined whether BMP3 and BMP7 affected the stability of the myofibroblast phenotype in culture. Treatment with BMP3 resulted in a significant reduction in the number of cells with αSMA stress fibers (Fig. 4c, d), which displayed uneven distribution of staining throughout the cytosol (Fig. 4c, BMP3 inset). In contrast, treatment with BMP7 did not reduce the number of cells with αSMA stress fibers compared to the induced myofibroblasts (Fig. 4c, d). We also tested whether BMP3 and BMP7 directly antagonize TGFβ2 induction of myofibroblasts by simultaneously treating corneal fibroblasts with either BMP3 or BMP7 during the induction step (Supplementary Fig. 5a). Under these conditions, neither BMP3 nor BMP7 impacted the formation of αSMA stress fibers (Supplementary Fig. 5b). These data indicate that TGFβ2 is a stronger inducer of myofibroblast differentiation, and that BMP3 promotes fiber disassembly, which contributes to the destabilization of the myofibroblast phenotype.

### BMP3 destabilizes αSMA stress fibers in myofibroblasts by promoting disassembly of focal adhesions

**Fig. 5:**
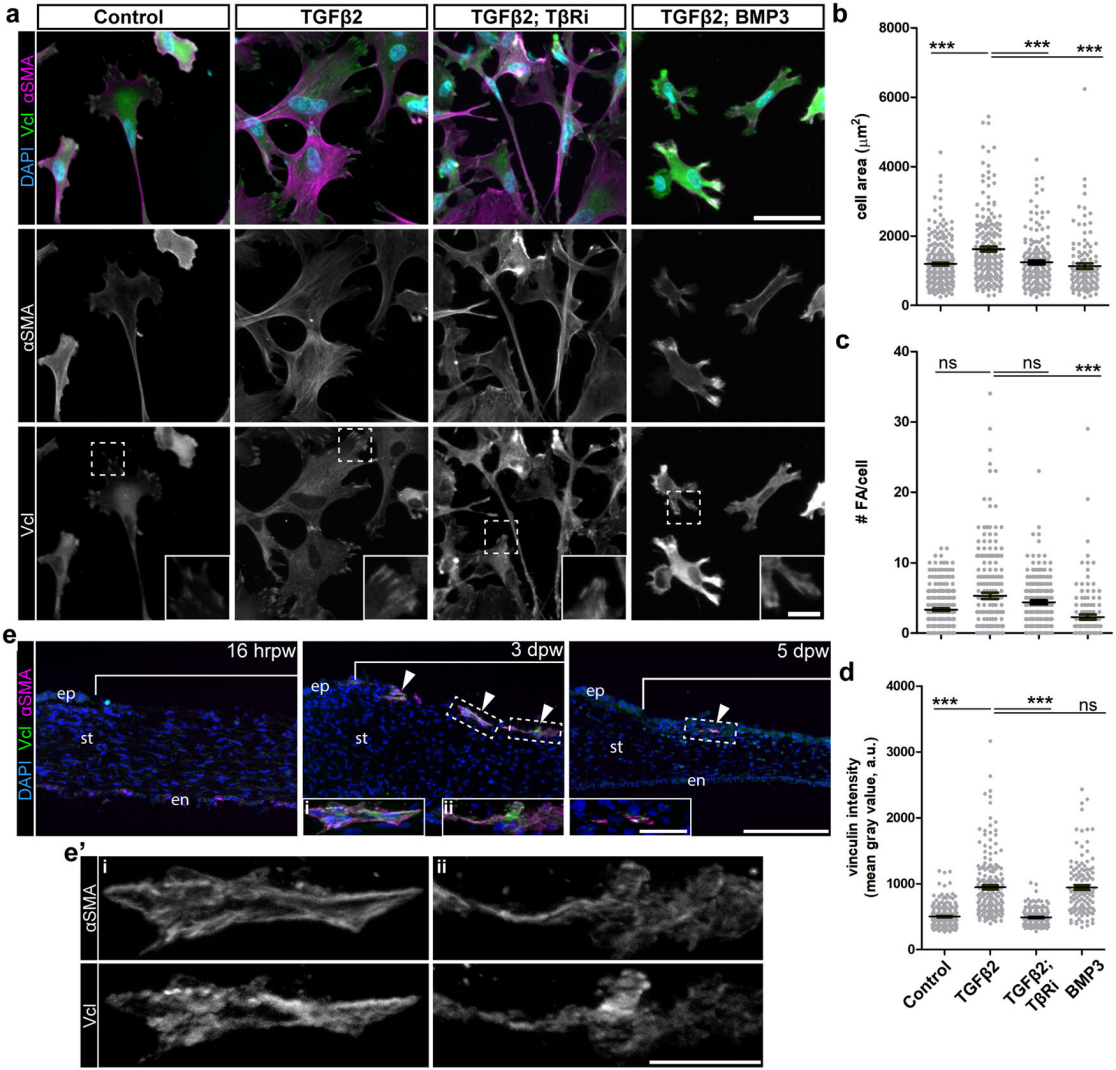
BMP3 promotes focal adhesion turnover and αSMA fiber disassembly. **a** Corneal fibroblasts were either cultured uninduced (control) or induced with TGFβ2 for 72 h. Cells were then either untreated or treated with TβRi, or BMP3 for 72 h. Samples were immunostained for αSMA (magenta) to identify myofibroblasts and vinculin (Vcl, green) to observe focal adhesions. **b**–**d** Quantification from (**a**) included (**b**) cell area, (**c**) number of focal adhesions per cell, and (**d**) total vinculin staining intensity per cell for each condition. (*N* = 3 independent experiments). **e** Histological sections of healing corneas from 16 hrpw, 3 dpw and 5 dpw corneas were immunostained for αSMA (magenta) and vinculin (Vcl, green). Colocalization of αSMA and Vcl was observed (arrowheads). **e'** High magnification of cells that appear to be under tension (**i**) or reorganizing αSMA and vinculin positive adhesion complexes (**ii**) within 3 dpw wounded cornea. Non-parametric One-way ANOVA with Kruskal–Wallis post-hoc test (**b**–**d**) was performed. ***p < 0.001, ns not significant. Scale bars: 25 µm. Scale bars: 50 µm, inset: 10 µm. Scale bars: 100 µm, inset: 25 µm.

Myofibroblast differentiation requires integration of TGFβ and focal adhesion signal transduction^2,41,87–89^. To test whether BMP3 prevents stability of the myofibroblast phenotype by disrupting focal adhesions, we treated TGFβ2-induced myofibroblasts with BMP3 (Fig. 5a) and immunostained for focal adhesion complexes using vinculin antibody. In TGFβ2-induced myofibroblasts, αSMA stress fibers terminated in vinculin-positive focal adhesions, which was less evident in uninduced control cells and in the presence of TβRi (Fig. 5a). In addition, the TGFβ2-induced myofibroblasts displayed many features of highly adherent cells, including increased cell spreading (Fig. 5b) and elevated vinculin staining (Fig. 5d), but the number of foci was not significantly changed compared to uninduced control cells (Fig. 5c). In contrast, induced cells treated with BMP3 were significantly smaller and displayed fewer focal adhesions compared to untreated myofibroblasts (Fig. 5b, c). Focal adhesion sizes were significantly reduced in BMP3 treated cells compared to TGFβ2-induced myofibroblasts (Supplementary Fig. 6a). Total vinculin staining intensity within BMP3 treated cells was unchanged from induced myofibroblasts (Fig. 5d), however, the protein distribution in BMP3 treated cells appeared largely cytosolic (Fig. 5a). These results indicate that BMP3 promotes disassociation of vinculin, which disrupts its accumulation in focal adhesions and destabilizes αSMA stress fibers.

Next, we speculated that if myofibroblasts are similarly regulated during wound healing of the embryonic cornea, then expression of vinculin would correlate with the transient expression of αSMA. Vinculin staining was not detected prior to αSMA expression in the anterior wound at 16 hrpw (Fig. 5e). However, by 3 dpw, αSMA-positive cells stained positive for vinculin (Fig. 5e, arrowheads). At higher magnification, some of the double-labeled cells reveal colocalization of αSMA stress fibers with vinculin in the sharply defined edges of pointed cell extensions (Fig. 5e; inset i; and 5e’). However, some αSMA-positive cells with low levels of vinculin staining exhibit less defined cell boundaries and fewer prominent cellular extensions (Fig. 5e; inset ii; and 5e’). By 5 dpw, very few αSMA-positive cells were detected in the wound and the residual staining correlated with low levels of vinculin protein (Fig. 5e; arrowhead and inset). Taken together, our results indicate that vinculin is exclusively expressed by αSMA-positive myofibroblasts in the wound, but it is reduced during re-epithelization of the cornea. Suggesting that TGFβ2 stimulates the myofibroblast phenotype via accumulation of vinculin focal adhesions, but BMP3 promotes their disassociation from the focal adhesion complex, which results in the unstable presence of myofibroblasts in the healing corneal wounds.

### BMP3 signals via ALK2/ALK3 to destabilize myofibroblast phenotype

**Fig. 6:**
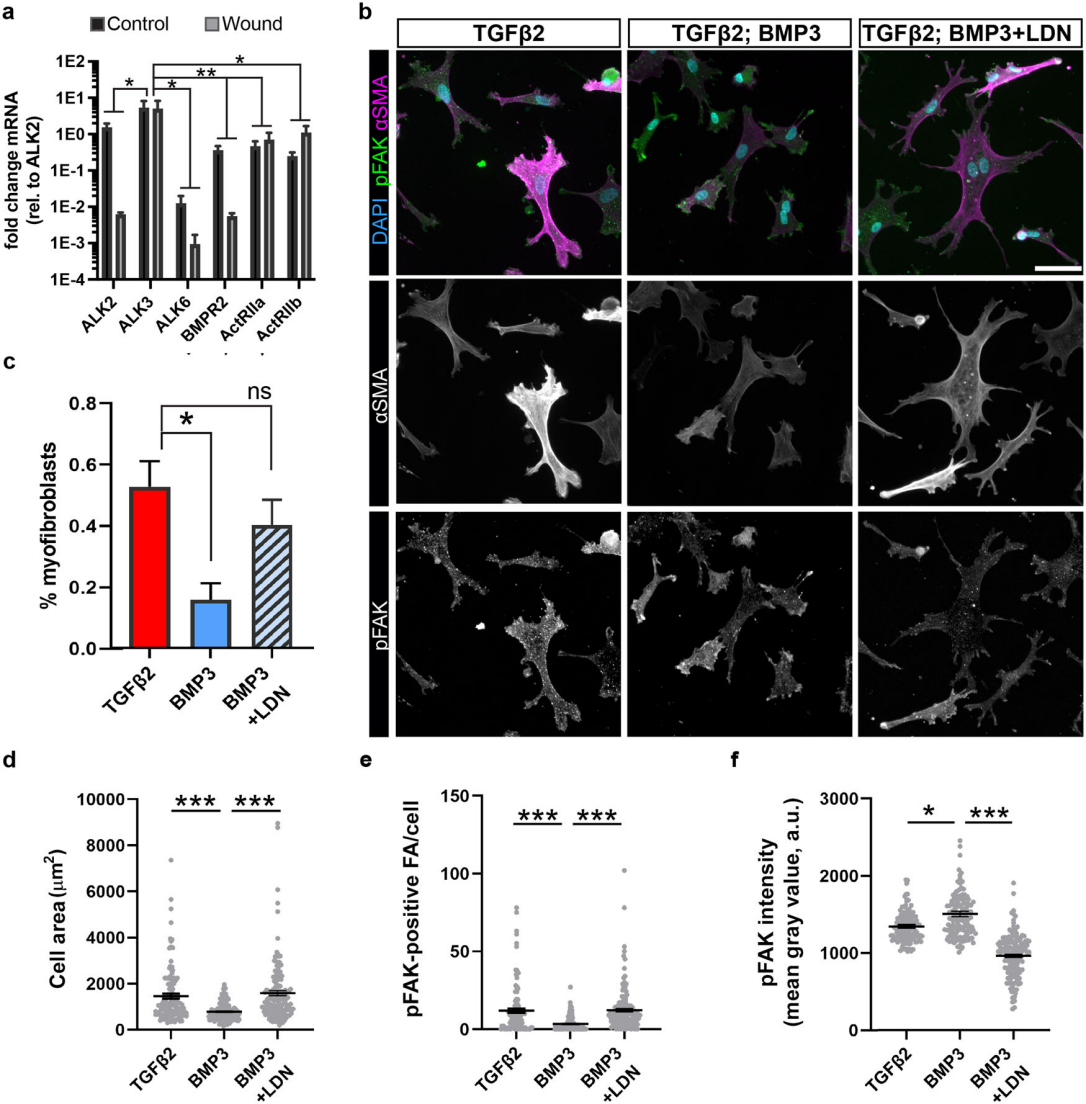
Inhibition of ALK2/ALK3 in the presence of BMP3 maintains TGFβ2-induced myofibroblast phenotype. **a** qPCR analysis of *ALK2*, *ALK3*, *ALK6*, *BMPR2*, *ActRII*, and *ActRIIB* transcript levels in 5 dpw wound and stage-matched control corneas showing significant enrichment of *ALK3* compared to other receptors (*N* = 3 independent samples). **b** TGFβ2-induced corneal fibroblasts were then either untreated or treated with BMP3 alone, or BMP3 in the presence of LDN-193189 for 72 h. Samples were immunostained for DAPI (blue) and αSMA (magenta) to identify myofibroblasts and pFAK (green) to observe focal adhesions. A reduction in myofibroblast phenotype was observed in cells treated with BMP3 alone, compared to the TGFβ2-induced and BMP3 in the presence of LDN-193189. **c** Quantification of the percentage of myofibroblasts observed in **b** (*N* = 3 independent experiments; images were taken from 5 fields of each sample and the number of cells averaged; *N* = 204 cells TGFβ2, *N* = 143 cells TGFβ2 + BMP3, and *N* = 235 cells TGFβ2 + BMP3 + LDN). Data for **a** and **c** were assumed to be normally distributed and are shown as mean ± SEM. For **a** Tow-way ANOVA with Bonferroni’s post-test was performed. For **c** One-way ANOVA with Tukey’s post-test was performed. *p < 0.05, **p < 0.01, ns not significant. **d**–**f** Quantification from **b** included: **d** cell area, **e** number of pFAK-positive focal adhesions per cell, and **f** total pFAK staining intensity per cell for each condition. (*N* = 3 independent experiments; images were taken from 5 fields of each sample and the number of cells averaged; *N* = 121 cells TGFβ2, *N* = 129 cells TGFβ2 + BMP3, and *N* = 147 cells TGFβ2 + BMP3 + LDN). Non-parametric One-way ANOVA with Kruskal–Wallis post-hoc test (**d**–**f**) was performed. *p < 0.05, ***p < 0.001. Scale bar 25 µm.

To understand the mechanism by which BMP3 signals during embryonic cornea wound healing, we compared the transcript levels of BMP receptors *ALK2*, *ALK3*, *ALK6*, *BMPR2*, *ActRII*, and *ActRIIB* between 5 dpw and stage-matched controls. qPCR analysis revealed no significant differences in the levels of expression of all receptors between wounded and control corneas, but ALK3 was significantly upregulated (Fig. 6a). We, therefore, sought to determine whether BMP3 signals via ALK2/ALK3 to destabilize TGFβ2 induced myofibroblast phenotype in cultured corneal fibroblasts. For this analysis, we utilized the small molecule LDN-193189, which inhibits BMP signaling by disrupting ALK2 and ALK3 receptors^90–92^. Our results show that induction of myofibroblasts was not inhibited in the presence of BMP3 and LDN-193189 (Fig. 6b, c). Furthermore, analysis of cell spreading showed that the cell area was significantly larger in the presence of LDN-193189 than in cells treated with BMP3 alone (Fig. 6d).

Next, we investigated the mechanism by which BMP3 inhibits the myofibroblast phenotype. Given that myofibroblast differentiation depends on integrin signaling and focal adhesion kinase (FAK)^93,94^, we examined the levels of active FAK signaling using the phosphorylated FAK (pFAK) antibody. Our results revealed that consistent with vinculin staining, TGFβ2 stimulates pFAK-positive focal adhesions and their number was significantly reduced in the presence of BMP3 alone, but not affected when cells were treated with BMP3 in the presence of LDN-193189 (Fig. 6b, e). Similarly, focal adhesion size was significantly reduced in the presence of BMP3 alone, but increased in the presence of BMP3 and LDN-193189 (Supplementary Fig. 6b). In addition, the levels of pFAK staining were significantly elevated in the cytosol of cells treated with BMP3 alone compared to TGFβ2 and BMP3 in the presence of LDN-193189 (Fig. 6f). Together, these results indicate that BMP3 signaling regulates pFAK-positive foci and myofibroblast differentiation through either ALK2 or ALK3.

## Discussion

Regulation of myofibroblast differentiation is a consistent attribute to non-fibrotic healing in embryonic wounds^70,95^. Although myofibroblast differentiation is endogenously regulated in embryonic cornea wounds^74^, the mechanistic regulation of this cell population is not fully understood. Here, we show that in the embryonic cornea, myofibroblasts are negatively regulated by the regenerating epithelium, probably due to cytokines that are sequestered in the epithelial basement membrane (EBM)^96–99^. At the molecular level, pSMAD2 was involved in the activation of myofibroblasts, and pSMAD1/5/8 was responsible for their transient phenotype. Transient activation of pSMAD2 is an intrinsic property of embryonic fibroblasts, as fetal dermal fibroblasts stimulated with TGFβ1 exhibit this phenotype, which is absent at post-natal stage^100^. Using the embryonic wound healing model, we mapped the spatiotemporal shift from TGFβ predominant to BMP dominated signaling in the wound and correlated this transition to re-epithelialization of the wound during non-fibrotic cornea repair. We inferred that TGFβ and BMP signal transduction within the cornea stroma is controlled by the healing cornea epithelium, which lead us to identify key ligands expressed in the model. Using a combination of focused transcriptional profiling, histological analysis, and in vitro culture of primary embryonic keratocytes, we identified differential expression of TGFβ2, BMP3, and BMP7 ligands and dissected how they regulate the myofibroblast transition in the healing embryonic cornea.

In adult cornea wounds, loss of the EBM permits entry of profibrotic cytokines into the stroma which contributes to myofibroblast induction^101,102^. Our data show that in the embryonic cornea wounds, myofibroblast induction and clearance occurs prior to complete re-epithelialization and that both *TGFβ1* and *TGFβ3* are expressed during this process. TGFβ1 is probably sequestered in the regenerating EBM, whereas the antifibrotic^103,104^ and non-heparin binding^105,106^ TGFβ3 is likely not concentrated in the EBM. Both isoforms are involved in regeneration of the corneal epithelium during the early phase of wound healing^107^. *TGFβ2* is robustly expressed in the developing stroma prior to myofibroblast differentiation. While the mechanism of TGFβ2 activation in healing embryonic corneas is unclear, mechanical retraction of the wound due to increase in the size of the growing eye may be sufficient to stimulate activation of pSMAD2. TGFβ is sequestered as an inactive form in the ECM by LTBP^108^. Given that *LTBP1* is highly expressed in the cornea stroma at E7^109^, it may play a role in in this process during development. Tissue culture models have revealed that mechanical stress on the ECM is sufficient to release TGFβ1 from this inactive, caged conformation^110^ which allows for activation of pSMAD2^111,112^. Thus, it is possible that the ECM in the cornea wound is laden with sequestered TGFβ2 prior to injury and wound retraction releases it in the anterior stroma. We cannot rule out other mechanisms of TGFβ activation in the corneal wound such as integrins^113,114^, matrix metalloproteinases^115^, reactive oxygen species^116,117^, and thrombospondin-1^118,119^.

The superficial localization of myofibroblast differentiation could be correlated to mechanical loading in the retracting anterior stroma^87,120–123^. Studies have shown that stretched substrates stimulate local TGFβ1 activation from LTBP, promoting the formation of αSMA stress fibers within myofibroblasts^124^. The αSMA fibers remain under tension due heightened cell adhesion and cell contraction, which further stabilizes the myofibroblast phenotype^2,87,125,126^. We showed that TGFβ2 stimulates the accumulation of αSMA stress fibers and mature focal adhesions in primary embryonic keratocytes. Similarly, TGFβ2 is released in the anterior stroma as the wound stretches until 3–4 dpw, which induces superficial myofibroblast differentiation. TGFβ2 is expressed in the corneal epithelium^127–129^. During wound healing, damage to the EBM exposes the stroma to TGFβ2, which together with paracrine signaling from the stromal cells induces myofibroblasts^130,131^. TGFβ2 and increased pSMAD2 staining was also observed in the corneal epithelium of samples obtained from patients with severe keratoconus^132^.

We found that *BMP3* and *BMP7* are localized in the regenerating epithelium of embryonic cornea wounds. It is likely that BMP3 and BMP7 ligands are sequestered in the EBM of the regenerating epithelium, which provides localized concentrations that activate pSMAD1/5/8 in the wounded stroma. BMP7 binds with either BMPRIIA or ActRII/IIb which heterodimerizes with ALK2 to stimulate pSMAD158 activation^53,133–136^. Expression pSMAD1/5/8 was induced upon TGFβ1treatment in corneal fibroblasts derived from normal and keratoconus corneas^137^. Indicating that pSMAD1/5/8 can also be induced via non-canonical pathway in adult corneal fibroblasts. Previous studies demonstrated that BMP7 has anti-fibrotic response in various models including renal fibrosis^32,56,138^, liver fibrosis^57^, dermal papilla cells^139^, and cornea wounds^67,68,94^. Most of what is known about BMP3 signal transduction is derived from studies in chondrogenic and osteogenic cell lineages. BMP3 can only bind ActRII/IIb^140–142^ and it is unclear which type I receptors it can activate. During bone development, BMP3 recruits ALK4 and ALK5 type I receptors, which results in the activation of pSMAD2, but it is unknown if BMP3 can activate pSMAD1/5/8 through ALK2 or ALK3, both of which are highly expressed in the developing embryonic cornea^109^. Structural analysis of BMP3 and putative BMP receptors reveal that BMP3 has high affinity for ActRIIb type II receptor and ALK3 type I receptor^143^, which potentiates BMP3 as a pSMAD1/5/8 activator. Both ActRIIb and ALK3 are expressed in the chick cornea^109^ and during wound healing, whereas inhibition of ALK2/ALK3 abrogated BMP3-induced reduction of myofibroblasts in vitro. Indicating that BMP3 could potentially activate pSMAD1/5/8 during development. In embryonic tissues, BMP3^140^ and BMP7^144^ strongly inhibit activation of pSMAD2. Given that they are both differentially upregulated in the healing embryonic cornea epithelium, raises the possibility that the proteins are sequestered in the regenerating EBM^145–147^ and contribute to activation of pSMAD1/5/8 and suppression of pSMAD2 in the anterior stroma of the embryonic cornea wound. In contrast with the adult cornea wounds where complete wound closure and regeneration of the EBM is critical for regulating myofibroblast survival^131,148,149^, we found that in the embryonic cornea wounds, myofibroblast induction and clearance occurs prior to complete re-epithelialization. One possibility is that the EBM of the regenerating epithelium is sufficient to sequester and release active BMP3, which regulates myofibroblast induction and prevents their persistence in the embryonic cornea wounds.

Overexpression of BMP7 antagonizes myofibroblast differentiation in adult corneal wounds via activation of pSMAD1/5/8 and suppression of myofibroblast related genes in mouse and rabbit cornea models^83,85,150^. One of the mechanisms of BMP7 signaling in the adult cornea wounds is through promotion of myofibroblast apoptosis^94^. This is unlikely to be the mechanism in the embryonic cornea where very few apoptotic cells occupy the wound and no increase was observed during the healing process^62^. BMP7 stimulates vinculin positive focal adhesions and does not prevent myofibroblast differentiation in cultured primary human adult keratocytes^82^, similar to our observation in TGFβ2-induced corneal fibroblasts. BMP3 suppresses TGFβ1-induced myofibroblast differentiation in murine pulmonary fibrosis models^151^. In human lung tissue, BMP3 serves as a prognostic tool to predict the level of lung fibrosis and patient survival rate, but the mechanism by which it regulates myofibroblast differentiation was not described. Based on our in vitro studies with the LDN-193189 inhibitor and pFAK staining, we conclude that BMP3 signals through ALK2/ALK3 to disrupt the TGFβ2-induced myofibroblast phenotype via FAK-dependent mechanism to destabilization of focal adhesions. This is likely to be dependent on integrin signaling, which has been shown to activate myofibroblasts^45,94^ and also prevent their apoptosis in response to profibrotic cytokines^152^.

**Fig. 7:**
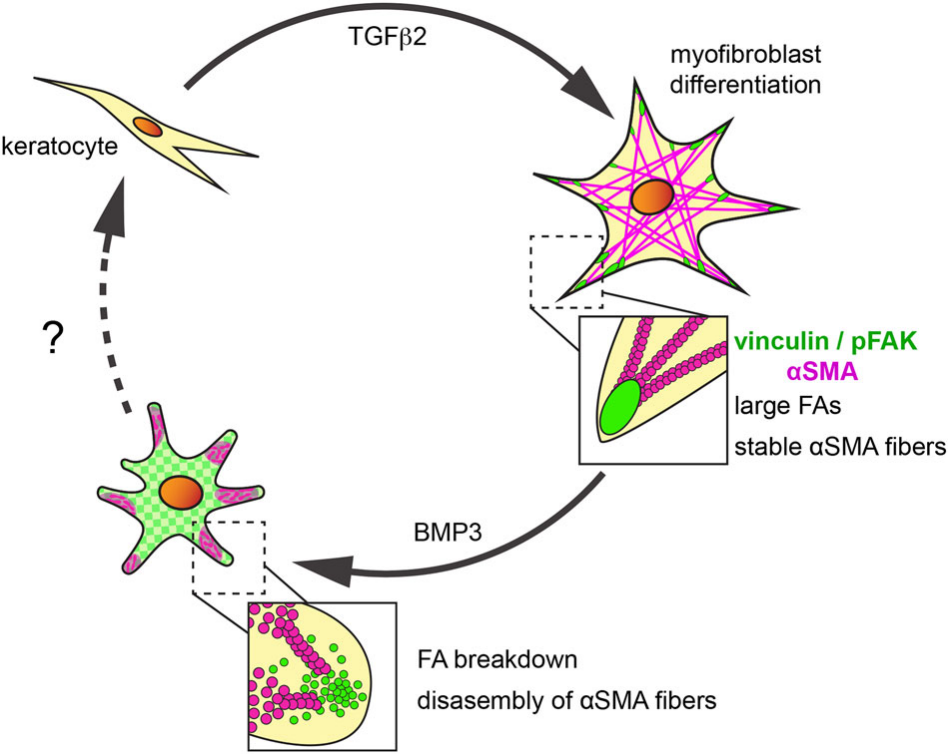
Transient myofibroblast phenotype is regulated by BMP3. Embryonic keratocytes stimulated with TGFβ2 differentiate into myofibroblasts, which is indicated by organization of αSMA into stress fibers that are anchored in focal adhesion complexes enriched with pFAK and vinculin. Treatment with BMP3 alters the myofibroblast phenotype by inducing turnover of pFAK and vinculin, and disassembly of αSMA fibers via ALK2/ALK3. This results in diffuse cytosolic localization of pFAK and vinculin and aggregates of αSMA in the cell cytosol. Under this state, it is possible that the cells revert to normal keratocyte phenotype and contribute to corneal transparency.

Our results showed that BMP3 negatively regulates TGFβ2-induced myofibroblast phenotype by disrupting focal adhesion stability (Fig. 7). During TGFβ2 treatment, corneal fibroblasts were transformed into myofibroblasts with numerous large focal adhesions that tethered αSMA stress fibers to adhesion sites (Figs. 5 and 7). Subsequent treatment with BMP3 promoted cytosolic accumulation of vinculin and pFAK, which facilitated disassembly of αSMA fibers into aggregates and loss of the myofibroblast phenotype (Figs. 5a, 6c, and 7). Given that vinculin co-localized with myofibroblasts and corresponded with the transient expression of αSMA in the embryonic cornea wounds, it is likely that TGFβ2 and BMP3 regulate this process in vivo via a similar mechanism. Since embryonic cornea wounds do not exhibit significant apoptosis associated with the healing cascade^74^ and have properly organized collagen structure when fully healed^75^, we posit that after myofibroblasts lose their phenotype, they revert back to keratocytes and complete cornea development (Fig. 7 dotted line). This work highlights a mechanism that targets myofibroblast adhesion to modulate their differentiation during non-fibrotic cornea wound healing and provides insights into potential candidates for therapeutic intervention to prevent fibrosis in adult tissues.

## Methods

### Animals

Fertilized White Leghorn chicken eggs (TAMU, College Station, TX) were incubated at 38 °C until needed. For cornea wound healing, embryos were accessed in ovo and wounded as previously described^74^. Briefly, corneas were wounded at E7 by making a linear incision with a micro-dissecting knife (Fine Science Tools, CA) across the cornea center, with lacerations traversing the corneal epithelium, basement membrane, and anterior stroma. The stage of corneal development at E7 in chicks would be equivalent to approximately E15.5 in mice^153^ and week 8 in humans^154^. All experiments with animals complied with ethical handling procedures approved by the Rice University Institutional Animal Care and Use Committee.

### Histology and immunostaining

Embryos were collected at 16 h, and at 3 and 5 days during the wound healing process. After decapitation, eyes were collected in Ringer’s solution and fixed overnight in 4% paraformaldehyde at 4 ˚C. All experiments were performed at least twice. The sample numbers in each experiment are reported in the Figure Legends. For whole-mount immunostaining, the region of the anterior eye was dissected and processed as previously described^155^. For sections, corneas were dissected from the eyes, dehydrated in ethanol series (50%, 70%, 90%, and 100%), infused with Histosol™ (Electron Microscopy Sciences), then embedded in paraffin. Corneas were sectioned at 10 μm thick and prepared for immunostaining using standard protocols. The following antibodies were used diluted in antibody buffer (phosphate buffered saline, PBS, containing 0.1% (v/v) Triton-X supplemented with 5% (v/v) heat inactivated goat serum and 0.1% bovine serum albumin): Mouse anti-α-SMA (1:400; IgG2a, Sigma, Cat# A2547) was diluted 1:400 to label myofibroblasts in tissue and 1:800 for cell culture analysis. Rabbit anti-pSMAD1/5/8 (1:200; IgG, Cell Signaling, Cat# 9516), rabbit anti-pSMAD2 (1:400; IgG, Cell Signaling, Cat# 18338), mouse anti-metavinculin (Vcl, 1:30; IgG1, Developmental Studies Hybridoma Bank, DHSB, Cat# VN 3–24), mouse anti-laminin (1:30; IgG1 DSHB, Cat# 31 or 31-2), and rabbit anti-FAK (pY397) was used to label activated FAK (1:200; IgG; Invitrogen, Cat# 44–624 G). Isotype and no primary antibody controls were performed for the immunostaining procedures. The following secondary antibodies (Invitrogen) were used at 1:200; Alexa-594 goat anti-mouse IgG2a, Alexa-488 goat anti-mouse IgG1, Alexa-488 goat anti-rabbit IgG. Whole-mount corneas and sections were counterstained with 4’,6-diamidino-2-phenylindole (DAPI) to label all nuclei. Slides were cover-slipped with Fluoromount-G^®^ (Southern Biotech) and fluorescent images were captured using a Zeiss Axiocam mounted on a Zeiss Axioskop 2 microscope.

### Confocal imaging of whole-mount corneas and tissue reconstruction

Following whole-mount immunostaining, corneas were cleared in glycerol and imaged with 20x objective on Nikon A1R with Nikon DS-Fi3 color camera. Imaris software was used to reconstruct stitched image stacks. The surface of wound occupied with myofibroblasts was quantified as a percentage of non-healed cornea wound using ImageJ software.

### In situ hybridization

Embryonic eyes were collected as described above and punctured in the posterior region of the globe to permit perfusion fixative. Samples were fixed overnight at 4 ˚C in modified Carnoy’s fixative (60% ethanol, 30% formaldehyde and 10% glacial acetic acid). Tissues were dehydrated in an ethanol series, embedded in paraffin, and sectioned at 10–12 µm thickness. Probes were PCR amplified from cDNA templates containing transcripts of the gene of interest and cloned into TOPO-II vector (Invitrogen). Primers used for amplifying the cDNA gene segment to clone into TOPO-II are listed in Supplementary Table 1. Anti-sense digoxygenin labeled riboprobes were generated from the TOPO-II clones by in vitro RNA transcription (DIG Labeling Kit, Roche). Section in situ hybridization was performed as previously described^156^.

### Quantitative PCR (qRT-PCR)

Healing corneas collected at desired time points were pooled for RNA extraction. Four corneas were pooled for 16 hrpw and two corneas were pooled for samples collected at 3 and 5 dpw. Contralateral uninjured corneas were used as stage-matched controls. Extraction of mRNA was conducted using TRIzol™ (Invitrogen), following manufacturer’s protocol. Residual genomic DNA was digested with Turbo™ DNA-*free™* kit (Invitrogen) and cDNA pools were generated using Verso cDNA Synthesis Kit (Thermo Scientific). qPCR was performed on a StepOnePlus Real-time PCR System (Applied Biosystems) using iTaq Universal SYBR Green SuperMix (Bio-Rad). Expression of each target gene was normalized to GAPDH. Primers used for reactions are listed in Supplementary Table 2.

### Recombinant proteins

Growth factors used to stimulate cells were active recombinant human proteins (Abcam). Lyophilized full-length h-TGFβ2 (ab84070), h-BMP3 (ab97412) and active BMP7 protein fragment (ab50100) were reconstituted in filter-sterilized water supplemented with 0.1% BSA to a concentration of 10 μg/mL, following manufacturer’s protocol. Media was treated for cell culture experiments at a concentration of 10 ng/mL for all growth factors.

### Isolation and culture of primary embryonic keratocytes

Fertilized eggs were incubated until E10 as described above. Embryos were decapitated and the heads were placed in Ringer’s saline solution supplemented with 100 U/mL penicillin and 100 µg/mL streptomycin (referred to at Ringer’s + P/S). Central squares of corneal tissue were dissected using microdissection scissors (item #: 15003-08; Fine Science Tools) and digested with filter sterilized dispase (1.5 mg/mL in DMEM + 10 mM HEPES; Worthington) for 5–10 min at 37 °C. Enzymatic digestion was quenched with Ringer’s solution supplemented with 0.1% BSA (Sigma-Aldrich). Fine forceps (item #: 11252-20, Fine Science Tools) were used to remove the epithelial layers and the endothelial layers were cut away using a micro-dissecting knife (item #: 10056-12; Fine Science Tools). Isolated stromal tissue was transferred to 0.25% collagenase (Worthington) in Ringer’s + P/S and incubated on a rotating plate at 37 °C for 1 h, or until tissue fragments were completely dissociated. The resulting cell suspension was passed through a 70 µm cell strainer (FisherBrand), washed with 5 mL of DMEM (Gibco) supplemented with 0.5% fetal bovine serum, 100 U/mL penicillin and 100 µg/mL streptomycin (referred to as complete DMEM). Cells were plated at 3 x 10^4^ cells/cm^2^ density on poly-L-lysine coverslips (72292-08; Electron Microscopy Sciences) precoated with 5 µg/cm^2^ rat tail collagen (Gibco) and allowed to attach for up to 24 h in complete DMEM with TGFβ receptor inhibitor SB-431542 (TβRi, 10 µM; Sigma). After cell attachment, culture media was replaced with complete DMEM supplemented with recombinant proteins or small molecule inhibitor for experimental conditions, including the ALK2/ALK3 inhibitor LDN 193189 (500 nM; Reprocell).

### Focal adhesion analysis

Focal adhesions were observed by staining cells for vinculin or pFAK, as described above. Segmentation of focal adhesions for analysis were conducted using ImageJ software, following image processing steps previously described^157^.

### Statistics

Each experiment was replicated at least three times and the number of samples analyzed are summarized in each figure legend. Statistical analyses were conducted using GraphPad Software. Data are presented as mean values with S.E.M. Differences between two means was determined by two-tailed unpaired Student’s *t*-test, while comparisons made between more than two means utilized one-way ANOVA followed by Tukey’s post-tests. Experiments that tested changes in gene expression across development stages were analyzed using two-way ANOVA followed by Bonferroni post-tests. Normal distribution was assumed for small sample sizes. Normality was tested for large sample sizes using the Shapiro–Wilk test and significance was determined using non-parametric methods. Statistics methods used are indicated in each figure legend. Results with *p* < 0.05 were considered significant.

### Reporting summary

Further information on research design is available in the Nature Research Reporting Summary linked to this article.

## Supplementary information

Supplementary Information

REPORTING SUMMARY

## Data Availability

The data supporting the findings in this study are available within the article and its Supplementary Information file. Any raw data generated and analyzed during this study are available from the corresponding author at request. Reasonable requests of unprocessed images and raw data files used for quantifications presented in the article or supplementary information may be submitted via email to lwigale@rice.edu.
