# Supplementary material for: BMP3 inhibits TGFβ2-mediated myofibroblast differentiation during wound healing of the embryonic cornea
Source: NPJ Regen Med. 2022 Jul 25;7:36. doi: 10.1038/s41536-022-00232-9 (PMC9314337; doi:10.1038/s41536-022-00232-9)
Supplement: Supplementary file 1 — Supplementary Information [file 41536_2022_232_MOESM1_ESM.pdf]

SUPPLEMENTARY FIGURES

Supplementary Figure 1

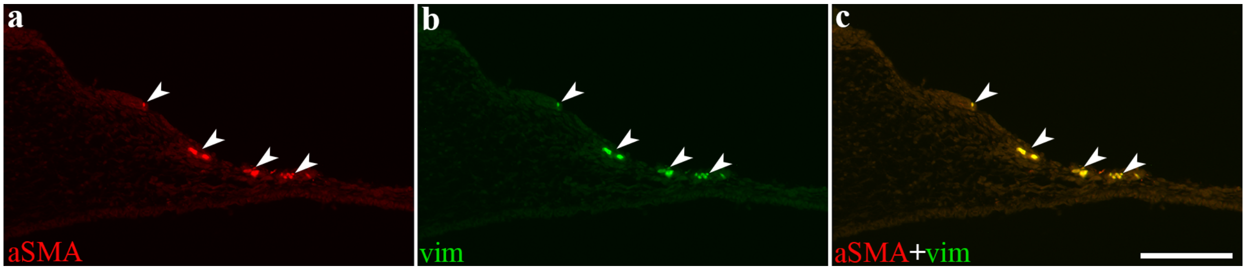

Expression of αSMA and vimentin (vim) during cornea wound healing. Immunohistological sections of 3 dpw corneas showing (**a**) αSMA (**b**) vim (**c**) colocalization of αSMA and vim (arrowheads). Mouse anti-vimentin IgG1antibody (AMF-17b, DHSB) was used at 1:40 dilution. Scale bar: 100 µm.

Supplementary Figure 2

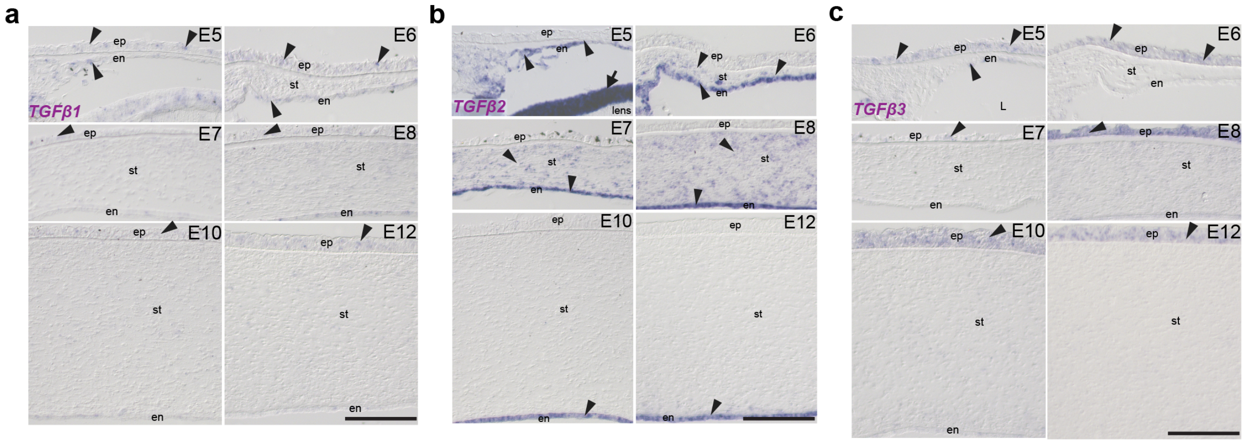

Expression of *TGFβ1*, *TGFβ2*, and *TGFβ3* during cornea development. Section *in situ* hybridization for: (**a**) *TGFβ1*, (**b**) *TGFβ2,* and (**c**) *TGFβ3* showing localization of mRNA (arrowheads) in the developing cornea between E5 to E12. Scale bars: 100 µm. Abbreviations: ep, epithelium; st, stroma; en, endothelium; L, lens.

Supplementary Figure 3

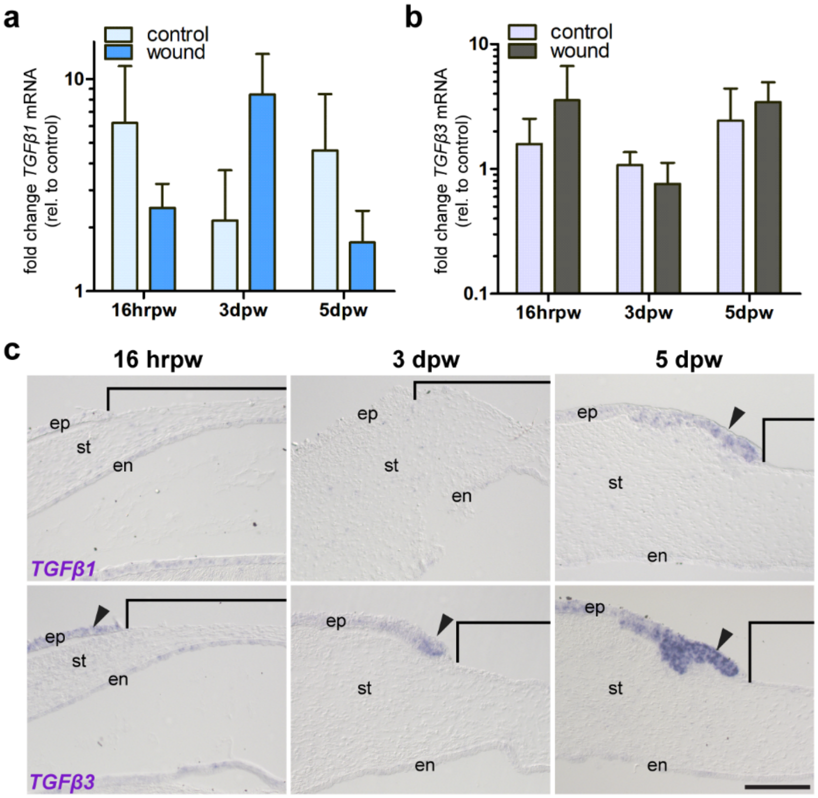

Expression of *TGFβ1* and *TGFβ3* during embryonic cornea wound healing. qPCR analysis of transcript levels of *TGFβ1* (**a**) and *TGFβ3* (**b**) in wounded embryonic corneas at 16 hrpw, 3 dpw and 5 dpw. Transcript levels were compared to stage matched controls for each respective timepoint (N = 4 independent samples for each timepoint). Data were assumed to be normally distributed and are shown as mean ± SEM. **c)** Section *in situ* hybridization showing localization (arrowheads) of *TGFβ1* and *TGFβ3* mRNA in wounded embryonic corneas at 16 hrpw, 3 dpw and 5 dpw. Scale bar: 100 µm. Abbreviations: ep, epithelium; st, stroma; en, endothelium; L, lens.

Supplementary Figure 4

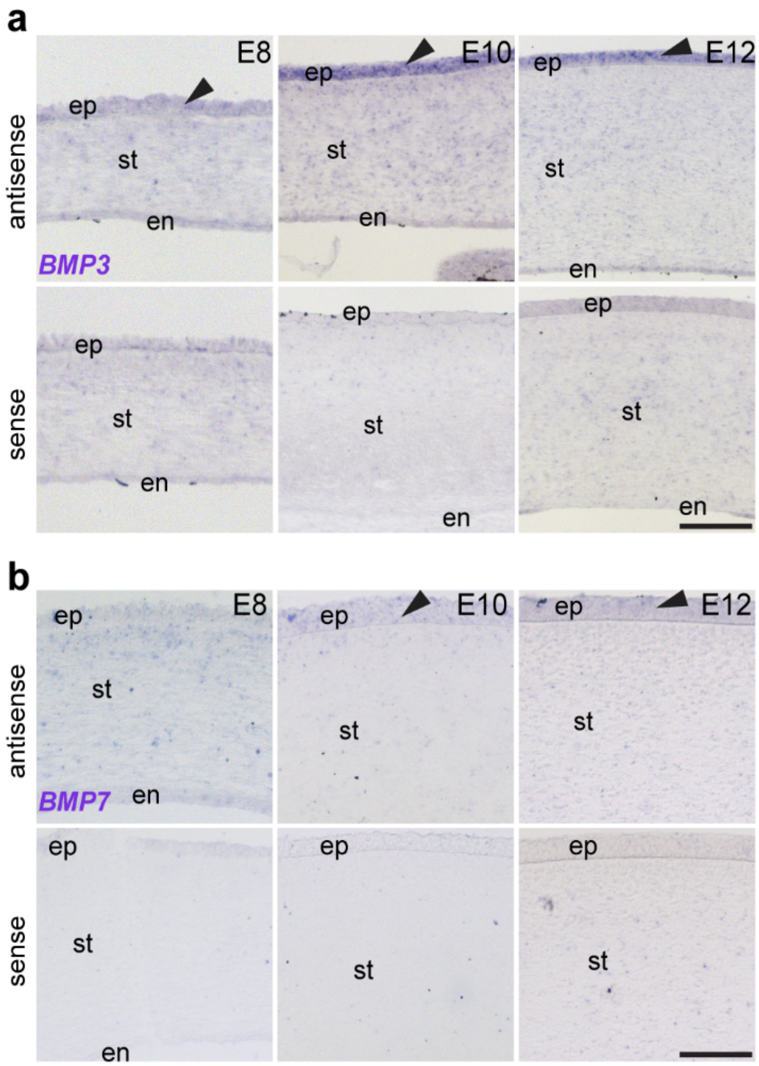

Expression of *BMP3* and *BMP7* during cornea development. Section i*n situ* hybridization showing localization of *BMP3* (**a**) and *BMP7* (**b**) mRNA (arrowheads) was performed on corneas from E8, E10 and E12 embryos. Due to low transcript levels in the embryonic corneas, sense riboprobes were used as control to identify non-specific staining of respective anti-sense riboprobe. Scale bars: 100 µm. Abbreviations: ep, epithelium; st, stroma; en, endothelium; L, lens.

Supplementary Figure 5

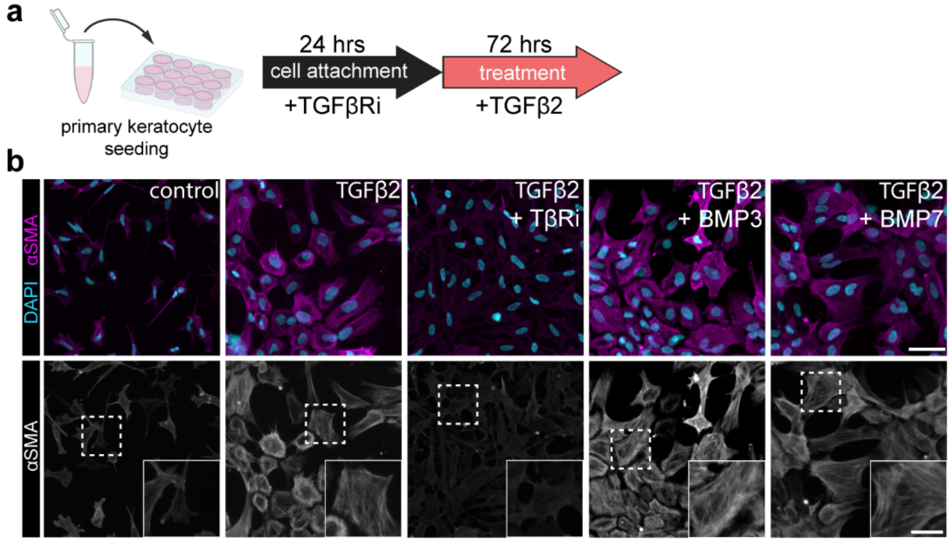

BMP3 and BMP7 do not directly inhibit embryonic myofibroblast differentiation. (**a**) Schematic of primary keratocyte isolation and simultaneous treatment of cells during myofibroblast induction (72 hours). (**b**) Cells treated with either TGFβ2, TGFβ2 and TGFβ receptor inhibitor SB-431542 (TβRi, 10 µM), TGFβ2 and BMP3, or TGFβ2 and BMP7 (each 10 ng/mL) were immunostained for αSMA and counterstained for DAPI to identify myofibroblasts. Scale bars: 50 µm, inset: 20 µm.

Supplementary Figure 6

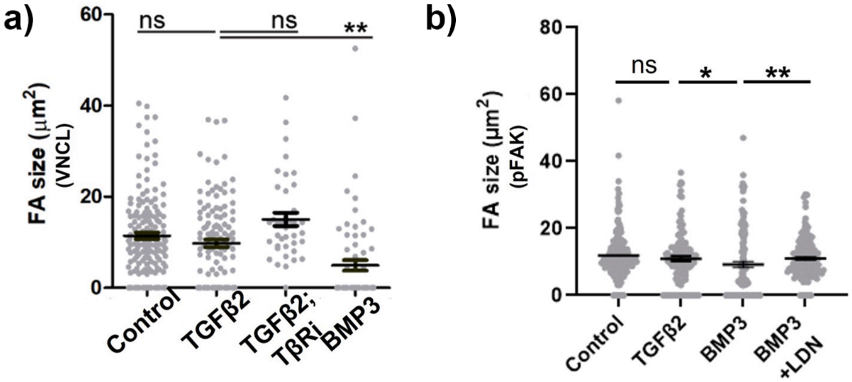

Quantification of the sizes of (**a**) vinculin-positive focal adhesion and (**b**) pFAK-positive foci under the indicated culture conditions. Treatment of TGFβ2-induced myofibroblasts with BMP3 resulted in significant reduction of vinculin-positive focal adhesion and pFAK-positive foci. Inhibition of BMP signaling restored the size of pFAK foci. (N = 3 independent experiments for **a** and **b**). Images were taken from 5 fields of each sample and the number of cells averaged; For **a**, N = 265 cells control, N = 196 cells TGFβ2, N = 73 cells TGFβ2+TβRi, N = 124 cells TGFβ2+BMP3. For **b**, N = 197 cells control, N = 121 cells TGFβ2, N = 129 cells TGFβ2+BMP3, N = 147 cells TGFβ2+BMP3+LDN. Non-parametric One-way ANOVA with Kruskal-Wallis post-hoc test (**d-f**) was performed. $^*p < 0.05$, $^{**}p < 0.01$.

SUPPLEMENTARY TABLES

Supplementary Table 1: Primer sequences used for riboprobe synthesis

Gene	Accession Number	Forward primer	Reverse Primer	Length	Hyb Temp
TGFβ1	NM_001318456	CAGCATCTTCTTCGTGTTCAAC	CAGCAGTTCTTCTCATCCGTC	528bp	66 ˚C
TGFβ2	NM_001031045	GTCATCTCCATCTACAACAGCAC	CTCTAAATCCTGGGACACGC	954bp	56.5 ˚C
TGFβ3	NM_205454	CAGAGAAGAACAGCACCAACC	ACAGCAGGGTGAAGCAGATG	719bp	62.5 ˚C
BMP3	NM_001034819	ACGCTGACTGGAACTGTT	CTCACTATGCTCTGGATGGTG	850bp	56.5 ˚C
BMP7	XM_417496	GTCGCCTTCTTCAAAGCC	AAGTATGTGTTGTGGGAGCC	744bp	61 ˚C

Supplementary Table 2: Primer sequences used for qPCR analysis

Gene	Accession Number	Forward Primer	Reverse Primer
TGFβ1	NM_001318456	TGGACCCGATGAGTATTGG	GTTGAACACGAAGAAGATGCTGT
TGFβ2	NM_001031045	GTTGTTACCCTCCTACAGACTT	ATGAATCCATTTCCAGCCA
TGFβ3	NM_205454	CTGGAGAGCCCAACACTG	CCTGTCGGAAGTCAATGTAAAGA
BMP3	NM_001034819	CTCAAGGTGGATTTTGCTGAC	CTCTCACTATGCTCTGGATGG
BMP4	NM_205237	CGGAGAAGAGGAGGAGAGC	TGCTGAGGTTGAAGACGAAG
BMP7	XM_417496	ACGCCAAAGAACCAGGAA	CACAGTAATACGCAGCATAGCC
ALK2	NM_001396493	GACAGTACATTGGCAGATTTATTGG	CCTCCAGACTTCTCCATAACG
ALK3	NM_001396493	ACGATTGTCAAGCAGAAGGG	ACGATTGTCAAGCAGAAGGG
ALK6	NM_001397879	TGTCTAGGATTAGAGGGCTCG	GCGTTGGGTGAAGATGTTTG
BMPR	NM_001001465	CGGAAACCATCCCACTTTTG	TTCAGCATCTTGATCCCAGC
ActRIIa	NM_205367	TCACGAAAAGGGTTCATTAACTGA	CTGAGCAATGTGACACAGCTCAT
ActRIIb	NM_204317	TCCCTGCTTAACATCCTGGTGTA	CCAAGAGGATGGCCACTGA
GAPDH	NM_204305	GATTCTACACACGGACACTT	CTGAGGGAGCTGAGATGATAAC
