# Supplementary material for: BMP3 inhibits TGFβ2-mediated myofibroblast differentiation during wound healing of the embryonic cornea
Source: NPJ Regen Med. 2022 Jul 25;7:36. doi: 10.1038/s41536-022-00232-9 (PMC9314337; doi:10.1038/s41536-022-00232-9)
Supplement: Supplementary file 2 — REPORTING SUMMARY [file 41536_2022_232_MOESM2_ESM.pdf]

nature portfolio

Corresponding author(s): Peter Lwigale

Last updated by author(s): Jun 7, 2022

Reporting Summary

Nature Portfolio wishes to improve the reproducibility of the work that we publish. This form provides structure for consistency and transparency in reporting. For further information on Nature Portfolio policies, see our Editorial Policies and the Editorial Policy Checklist.

Statistics

For all statistical analyses, confirm that the following items are present in the figure legend, table legend, main text, or Methods section.

n/a	Confirmed	
☐	☒	The exact sample size (*n*) for each experimental group/condition, given as a discrete number and unit of measurement
☐	☒	A statement on whether measurements were taken from distinct samples or whether the same sample was measured repeatedly
☐	☒	The statistical test(s) used AND whether they are one- or two-sided<br>*Only common tests should be described solely by name; describe more complex techniques in the Methods section.*
☐	☒	A description of all covariates tested
☐	☒	A description of any assumptions or corrections, such as tests of normality and adjustment for multiple comparisons
☐	☒	A full description of the statistical parameters including central tendency (e.g. means) or other basic estimates (e.g. regression coefficient) AND variation (e.g. standard deviation) or associated estimates of uncertainty (e.g. confidence intervals)
☐	☒	For null hypothesis testing, the test statistic (e.g. *F*, *t*, *r*) with confidence intervals, effect sizes, degrees of freedom and *P* value noted<br>*Give P values as exact values whenever suitable.*
☒	☐	For Bayesian analysis, information on the choice of priors and Markov chain Monte Carlo settings
☐	☒	For hierarchical and complex designs, identification of the appropriate level for tests and full reporting of outcomes
☒	☐	Estimates of effect sizes (e.g. Cohen's *d*, Pearson's *r*), indicating how they were calculated

Our web collection on statistics for biologists contains articles on many of the points above.

Software and code

Policy information about availability of computer code

Data collection	Images of focal adhesions were analyzed and processed using ImageJ software following previously published codes (https://doi.org/10.1016/j.mex.2014.06.004)
Data analysis	Graphpad

For manuscripts utilizing custom algorithms or software that are central to the research but not yet described in published literature, software must be made available to editors and reviewers. We strongly encourage code deposition in a community repository (e.g. GitHub). See the Nature Portfolio guidelines for submitting code & software for further information.

Data

Policy information about availability of data

All manuscripts must include a data availability statement. This statement should provide the following information, where applicable:

- Accession codes, unique identifiers, or web links for publicly available datasets
- A description of any restrictions on data availability
- For clinical datasets or third party data, please ensure that the statement adheres to our policy

The data supporting the findings in this study are available within the manuscript and its Supplementary Information file. Any data generated and analyzed during this study are available from the corresponding author at request.

Field-specific reporting

Please select the one below that is the best fit for your research. If you are not sure, read the appropriate sections before making your selection.

☒ Life sciences ☐ Behavioural & social sciences ☐ Ecological, evolutionary & environmental sciences

For a reference copy of the document with all sections, see nature.com/documents/nr-reporting-summary-flat.pdf

Life sciences study design

All studies must disclose on these points even when the disclosure is negative.

Sample size	No sample size calculation was used. Sample size was chosen to be between N = 3 to 7 depending on the variation found in the samples to find a reliable mean.
Data exclusions	No data was excluded.
Replication	All experiments were completed at least twice. Experiments that required quantitative analysis were independently repeated at least three times.
Randomization	Sample collection was random.
Blinding	Blinding was not possible in this study because the experimentalist carried out all the steps of sample collection through data analysis.

Reporting for specific materials, systems and methods

We require information from authors about some types of materials, experimental systems and methods used in many studies. Here, indicate whether each material, system or method listed is relevant to your study. If you are not sure if a list item applies to your research, read the appropriate section before selecting a response.

Materials & experimental systems

n/a	Involved in the study	
☐	☒	Antibodies
☒	☐	Eukaryotic cell lines
☒	☐	Palaeontology and archaeology
☐	☒	Animals and other organisms
☒	☐	Human research participants
☒	☐	Clinical data
☒	☐	Dual use research of concern

Methods

n/a	Involved in the study	
☒	☐	ChIP-seq
☒	☐	Flow cytometry
☒	☐	MRI-based neuroimaging

Antibodies

Antibodies used	Mouse anti-aSMA (Sigma, cat# A2547); rabbit anti-pSMAD2 (Cell Signaling, Cat# 18338); rabbit anti-pSMAD158 (Cell Signaling, Cat# 9516); rabbit anti-FAK pY397 (Invitrogen, Cat#44-624G); mouse anti-laminin (DSHB, Cat# 31 or 31-2); mouse anti-metavinculin (DHSB, Cat# VN3-24); Mouse anti-vimentin (DHSB, Cat# AMF-17b)
Validation	Specificity of the pSMAD2 and pSMAD158 antibodies was validated using isotype control. The aSMA,laminin, metavinculin, vimentin, and pFAKpY397 antibodies were validated from previous studies and specific staining of other ocular tissues and cells.

Animals and other organisms

Policy information about studies involving animals; ARRIVE guidelines recommended for reporting animal research

Laboratory animals	Fertilized chick eggs were obtained from commercial vendors. Chick embryos were used between embryonic day(E)7 and E12, prior to sex determination.
Wild animals	n/a
Field-collected samples	n/a
Ethics oversight	Rice University IACUC protocol number 20-190-RU

Note that full information on the approval of the study protocol must also be provided in the manuscript.
